# The plant membrane surrounding powdery mildew haustoria shares properties with the endoplasmic reticulum membrane

**DOI:** 10.1093/jxb/erx403

**Published:** 2017-12-09

**Authors:** Mark Kwaaitaal, Mads Eggert Nielsen, Henrik Böhlenius, Hans Thordal-Christensen

**Affiliations:** Section for Plant and Soil Science, Department of Plant and Environmental Sciences, Copenhagen Plant Science Center (CPSC), Faculty of Science, University of Copenhagen, Denmark

**Keywords:** Barley organelle markers, endoplasmic reticulum (ER), extrahaustorial membrane (EHM), powdery mildew, Rab GTPase, Sar1 GTPase, secretion

## Abstract

Many filamentous plant pathogens place specialized feeding structures, called haustoria, inside living host cells. As haustoria grow, they are believed to manipulate plant cells to generate a specialized, still enigmatic extrahaustorial membrane (EHM) around them. Here, we focused on revealing properties of the EHM. With the help of membrane-specific dyes and transient expression of membrane-associated proteins fused to fluorescent tags, we studied the nature of the EHM generated by barley leaf epidermal cells around powdery mildew haustoria. Observations suggesting that endoplasmic reticulum (ER) membrane-specific dyes labelled the EHM led us to find that Sar1 and RabD2a GTPases bind this membrane. These proteins are usually associated with the ER and the ER/*cis*-Golgi membrane, respectively. In contrast, transmembrane and luminal ER and Golgi markers failed to label the EHM, suggesting that it is not a continuum of the ER. Furthermore, GDP-locked Sar1 and a nucleotide-free RabD2a, which block ER to Golgi exit, did not hamper haustorium formation. These results indicated that the EHM shares features with the plant ER membrane, but that the EHM membrane is not dependent on conventional secretion. This raises the prospect that an unconventional secretory pathway from the ER may provide this membrane’s material. Understanding these processes will assist future approaches to providing resistance by preventing EHM generation.

## Teaser

The plant-derived membrane surrounding the powdery mildew intracellular feeding structure, called the extrahaustorial membrane, accumulates the ER- and ER/*cis*-Golgi-associated Sar1 and Rab1 GTPases and stains with dyes for the ER.

## Introduction

Many microbes of major importance are extremely intimate with higher organisms as they live entirely or partially within the boundaries of their host’s cells, where they take advantage of a continuous nutrient supply. This is also true for several clades of filamentous fungi and oomycetes that interact with plants, either pathogenically or symbiotically. These microbes include the beneficial mycorrhizal fungi, the oomycete pathogens causing late blight, downy mildew and white rust, and the rust and powdery mildew fungi. In host plant cells, specialized fungal structures, called arbuscules or haustoria, are formed. These are directly connected to hyphae outside the cells. As haustoria and arbuscules grow, the plant cells generate a membrane around them, which allows the invader and the host to have an intimate coexistence for an extended period of time. These periarbuscular and extrahaustorial membranes (EHMs) separate the microbe from the host cytosol and mediate nutrient absorption and transfer of virulence factors into the host cytosol (Szabo & Bushnell, 2001; O’Connell & Panstruga, 2006). Despite their obvious importance, the nature and source of these membranes remain unclear.

In plant cells, three major membrane trafficking pathways can be defined. These are the secretory pathway, the endocytic pathway and the vacuolar pathway (Kanazawa and Ueda, 2017). When *Ustilago maydis* attacks maize cells, the membranes surrounding the pathogen’s intracellular hyphae are labelled by plasma membrane (PM) markers (Djamei & Kahmann, 2012). This suggests that the PM is invaginated and/or that the endocytic pathway contributes to this membrane. Also, a number of PM-resident proteins and the marker for endocytosed membranes, FM4-64, co-localize with the EHM around haustoria of the oomycetes *Hyaloperonospora arabidopsidis* and *Phytophthera infestans* (Lu *et al.*, 2012). Furthermore, the EHM in *P. infestans*-invaded *Nicotiana benthamiana* cells is labeled by the multivesicular body/tonoplast-localized GTPase *Nb*RabG3c (Bozkurt *et al.*, 2015). This suggests endocytic delivery via multivesicular bodies (MVBs) is involved in the formation of the EHM when plants are infected by these two oomycete pathogens.

Several PM markers failed to accumulate on the Arabidopsis EHM formed around *Golovinomyces cichoracearum* powdery mildew haustoria (Koh *et al.*, 2005). This indicates that either the EHM formation in response to *G. cichoracearum* does not involve endocytosis from the PM or that a filtering process excludes PM-targeted proteins from the EHM. The EHMs in cells invaded by powdery mildew as well as by rust fungal species are separated from the host PM by a neck band (Manners & Gay, 1977; Szabo & Bushnell, 2001; Larous *et al.*, 2008), which likely limits mixing of proteins and lipids between the membranes. A number of observations for the EHM around powdery mildew haustoria indicate that a conventional membrane trafficking pathway does not generate this membrane. Firstly, the EHM in the Arabidopsis–*G. cichoracearum* interaction is targeted by the resistance protein RPW8.2 in a vesicle-associated membrane protein (VAMP) 721/VAMP722-dependent process (Wang *et al.*, 2009; Kim *et al.*, 2014; Kuhn et al., 2016). These vesicle *N*-ethylmaleimide sensitive factor attachment protein receptors (v-SNAREs) are suggested to be involved in secretion by mediating vesicle fusions at the PM (Kwon *et al.*, 2008; Kuhn et al., 2016), and therefore the data suggest a secretory pathway to the EHM. Secondly, application of the fungal toxin brefeldin A (BFA) affects neither RPW8.2 delivery to the EHM (Berkey *et al.*, 2017) nor haustorial success rate (Nielsen *et al.*, 2012). The latter indirectly shows that EHM formation is insensitive to BFA. BFA blocks recycling of PM material to the site of attack via the *trans*-Golgi network (TGN) and MVBs (Nielsen *et al.*, 2012; Nielsen and Thordal-Christensen, 2013). Therefore, these BFA studies do not conflict with the existence of a secretory pathway to the EHM. Thirdly, Berkey *et al.* (2017) showed that FM4-64 does not reach the EHM following endocytosis. In plants, the TGN acts as an early endosome and a major sorting station for secretion, recycling, and trafficking to the vacuole via MVBs, and all these compartments readily stain with FM4-64 (Viotti *et al.*, 2010; Richter *et al.*, 2014). Therefore, the TGN is not the likely source of membrane delivery to the EHM, suggesting that neither secretion nor conventional vacuolar trafficking is involved in its formation. This conflicts with the fourth observation, namely that Rab5 GTPases associate with the EHM around powdery mildew haustoria in both the Arabidopsis–*G. orontii* and in the barley–*Blumeria graminis* f.sp. *hordei* (*Bgh*) interactions (Inada *et al.*, 2016). Plant Rab5 GTPases are suggested to be recruited to the TGN and follow the MVBs during their maturation (Singh *et al.*, 2014). Combined, these observations suggest an unconventional membrane trafficking route to the EHM.

While these observations do not allow conclusions to be drawn as to the likely membrane contributor to EHM formation, the close physical association of the endoplasmic reticulum (ER) tubules with the EHM (Harder *et al.*, 1978; Leckie *et al.*, 1995; Koh *et al.*, 2005; Micali *et al.*, 2011; Zhang *et al.*, 2013) could suggest vesicle exchange from the ER membrane to the EHM. This is supported by data showing the ER-membrane dye DiOC_6_ labelling the EHM of powdery mildew haustoria in pea (Leckie *et al.*, 1995). In fact, we show here that the EHM surrounding the haustoria of *Bgh* has ER-like properties. Our data suggest that it can be stained by ER-membrane dyes, and we find that two small GTPases, Sar1 and RabD2a, which are essential for COPII- and COPI-mediated vesicle trafficking between the ER and Golgi, are specifically associated with it. However, the EHM forms despite inhibition of conventional ER–Golgi trafficking after expression of dominant-negative versions of Sar1a and RabD2a. This leads us to suggest that the EHM obtains its ER-like properties from an unconventional trafficking pathway transporting membrane material from the ER.

## Materials and methods

### Plant and fungal growth

For this study, barley (*Hordeum vulgare*) cv. Golden Promise seedlings, grown at 20 °C with 16 h light (150 μE s^−1^ m^−2^) and 8 h dark, were used. *Bgh* (isolate DH14) was propagated by weekly inoculum transfer onto 1-week-old barley seedlings.

### Particle bombardment

The first leaves of 7-day-old seedlings were transiently transformed by particle bombardment as described by Douchkov *et al.* (2005). For marker localization studies in powdery mildew-infected cells, the leaves were inoculated 2 h after bombardment. The leaves were examined by microscopy from 2 d after inoculation (dai).

### Constructs and primers

The coding regions of *HvSar1a*, *HvSar1b* and *HvSar1c*, *HvRabD2a* and *HvRabG3b*, Genbank accessions AK252291.1, AK252372.1, AK250294.1, AK355333.1 and AK368361.1, respectively, were amplified using Gateway^®^ compatible primers. The coding regions of *HvERD2* and *HvSec12*, Genbank accessions AK250768.1 and AK356828.1, respectively, were amplified using two sets of cDNA specific primers. However, since AK356828.1 is a partial sequence, we designed the *Sec12* forward primer (see Supplementary Table S1 at *JXB* online) based on the barley *Sec12* genomic sequence and positioned it upstream from the start codon. See Supplementary Figure S1 for alignments of the encoded proteins to the closest Arabidopsis homologues. Subsequently, on the purified PCR product, a second PCR using Gateway^®^ compatible primers was performed. The coding sequences were amplified from barley Golden Promise cDNA. With a Gateway^®^ BP reaction (Invitrogen) the fragments were subsequently cloned into the pDONR201 donor vector. The dominant-negative mutations *Hv*Sar1a-T34N and *Hv*RabD2a-N121I were introduced using overlapping primers encoding the planned mutation using the Quickchange XL mutagenesis kit (Stratagene). Primer sequences can be found in Table S1. After validation of the donor constructs by sequencing, the inserts were transferred into the destination vector with a Gateway^®^ LR reaction (Invitrogen). The pUbi-mYFP-Gateway, pUbi-mCherry-Gateway, pUbi-Gateway-mYFP and pUbi-Gateway-mCherry destination vectors used in this study are described in Kwaaitaal *et al.* (2010), and the 35S-driven overexpression destination vectors p2FGW7 and p2WFHB-Gateway-GFP are described in Karimi *et al.* (2002) and Böhlenius *et al.* (2010), respectively. Table 1 lists the marker constructs used in this work.

**Table 1.** Markers used in this study

Marker/construct	Labels	Function	Reference
DiOC_6_	ER membrane	—	—
ER-Tracker Blue-White	ER membrane	—	—
Hexyl rhodamine B	ER membrane	—	—
SP–YFP–HDEL	ER lumen	—	Irons *et al.* (2003)
SP–mCherry–HDEL	ER lumen	—	Nelson *et al.* (2007)
*Hv*MLO–mCherry	Plasma membrane	Powdery mildew susceptibility	Kwaaitaal *et al.* (2010)
*Hv*ERD2–mCherry	ER membrane	HDEL receptor	Kwaaitaal *et al.* (2010)
*Hv*LTP–mCherry	Plasma membrane	Lipid transfer protein	Kwaaitaal *et al.* (2010)
ST–YFP	Golgi	52-amino acid signal anchor of sialyltransferase (from rat)	Brandizzi *et al.* (2002)
GFP–*Hv*RabG3b	Tonoplast/cytosol	GTPase, membrane fusion	This work
*Hv*Sar1a–mCherry	ER membrane/cytosol	GTPase, vesicle budding	This work
*Hv*Sar1a–mYFP	ER membrane/cytosol	GTPase, vesicle budding	This work
*Hv*Sar1b–mCherry	ER membrane/cytosol	GTPase, vesicle budding	This work
*Hv*Sar1c–mYFP	ER membrane/cytosol	GTPase, vesicle budding	This work
*Hv*Sec12–mCherry	ER membrane/cytosol	GEF for Sar1 activation	This work
GFP and mYFP–*Hv*RabD2a	ER/*cis*-Golgi/cytosol	GTPase, membrane fusion	This work
mCherry–*Hv*RabD2a	ER/*cis*-Golgi/cytosol	GTPase, membrane fusion	This work
*Hv*Sar1aT34N–mYFP (GDP-locked)	ER membrane/cytosol	Inhibition of vesicle budding	This work
*Hv*Sar1aT34N–mCherry (GDP-locked)	ER membrane/cytosol	Inhibition of vesicle budding	This work
Golgi–mCherry	Golgi	α-1,2-Mannosidase I (soybean)	Nelson *et al.* (2007)
CFP–YFP	Cytosol	—	Bethke *et al.* (2009)
*Hv*RabD2aN121I (nucleotide-free)	ER/*cis*-Golgi/cytosol	Inhibition of membrane fusion	This work
*Hv*ARA6–GFP	*trans*-Golgi network/MVB/cytosol	GTPase, MVB maturation	Inada *et al.* (2016)

### Confocal microscopy

Barley leaves with transformed epidermal cells were vacuum infiltrated with 0.01% Tween20 and subsequently mounted in the same buffer under a coverslip for confocal microscopy. Leica SP5-X, SP5-II and SP8 confocal laser scanning microscopes mounted with ×63 water immersion lenses with a numerical aperture of 1.2 were used. For detection and localization of the fluorophores, green fluorescent protein (GFP) was excited at 488 nm and detected between 500 and 540 nm, monomeric yellow fluorescent protein (mYFP) was excited at 514 nm and detected between 528 nm and 560 nm, while mCherry was excited at 543 nm (Leica SP5-II) or 587 nm using the super continuum white laser (Leica SP5-X) and detected between 600 and 640 nm. To limit signal bleed-through between channels, the measurements of each fluorophore were performed in independent tracks exciting only one fluorophore at a time.

### Staining with the ER-specific dyes

The dyes, ER-Tracker^tm^ Blue-White, DiOC_6_ and hexyl rhodamine B (all Invitrogen; Table 1), were vacuum infiltrated into 1 cm barley leaf pieces in 0.01% Tween20 at final concentrations of 5, 10, and 1.6 μM, respectively. After incubation for 30 min, excess dye was removed by a short wash in 0.01% Tween20 and afterwards mounted in the same buffer for microscopy. ER-Tracker Blue-White was excited with a UV laser at 405 nm and the fluorescence was detected between 420 and 540 nm. DiOC_6_ was excited at 488 nm and the fluorescence was detected between 495 and 520 nm. Hexyl rhodamine B was excited at 543 nm and the fluorescence was detected between 580 and 640 nm.

### Quantification of powdery mildew fungal infection efficiency

To determine the role of *Hv*Sar1a in powdery mildew haustorial establishment, barley leaf epidermal cells were transformed, using particle bombardment, with a p35S–mCherry construct as transformation reporter and equimolar amounts of pUbi–YFP (control), pUbi–*Hv*Sar1a or pUbi–*Hv*Sar1aT34N (GDP-bound). To determine the role of *Hv*RabD2a, transformation was made with a β-glucuronidase (GUS) reporter construct (pUbiGUS) mixed in equimolar amounts with p2WF7HB (empty vector control) or p35S–HvRabD2aN121I (nucleotide-free). One day after transformation, the leaves were inoculated with powdery mildew spores at a density of 200 spores per square millimeter. Two days later, the total number of transformed cells was determined either by the mCherry signal using a standard epifluorescence microscope, or by GUS staining using a standard light microscope. At the same time the number of transformed cells containing a haustorium was scored. For additional details, see Böhlenius *et al.* (2010). The haustorial index was calculated as the fraction of the transformed cell that hosted a haustorium relative to the same fraction in the control treatment. Three independent biological replicates were performed with two technical replicates in each experiment.

### Fluorescence redistribution after photobleaching

Fluorescence redistribution after photobleaching (FRAP) measurements were performed on epidermal cells 2 d after transformation with fluorescent constructs and inoculation with *Bgh*. For each FRAP measurement, an area of 4 × 4 μm positioned at a haustorial finger was selected. The fluorescent signal in this image area was initially recorded by scanning five times. Thereafter, the area was bleached with high excitation laser power after which the time-lapse acquisition was continued for 60 s to be able to follow and quantify fluorescence recovery in the bleached area. The recovery curves were evaluated and fit using the LAS AF lite software package (Leica Microsystems CMS GmbH) and Igor Pro 6 with the K_FRAPcalc v9i procedure developed by Kota Miura (EMBL, https://cmci-embl.gitlab.io/docs/analysis/frapcalc/, last accessed 15 November 2017). The values were normalized using Phair’s double normalization and fit with a model taking two diffusing species into account (Phair *et al.*, 2004). An average diffusion coefficient was calculated taking the relative fractions of the two diffusing species into account.

### Image processing and analysis

**Fig. 1.**
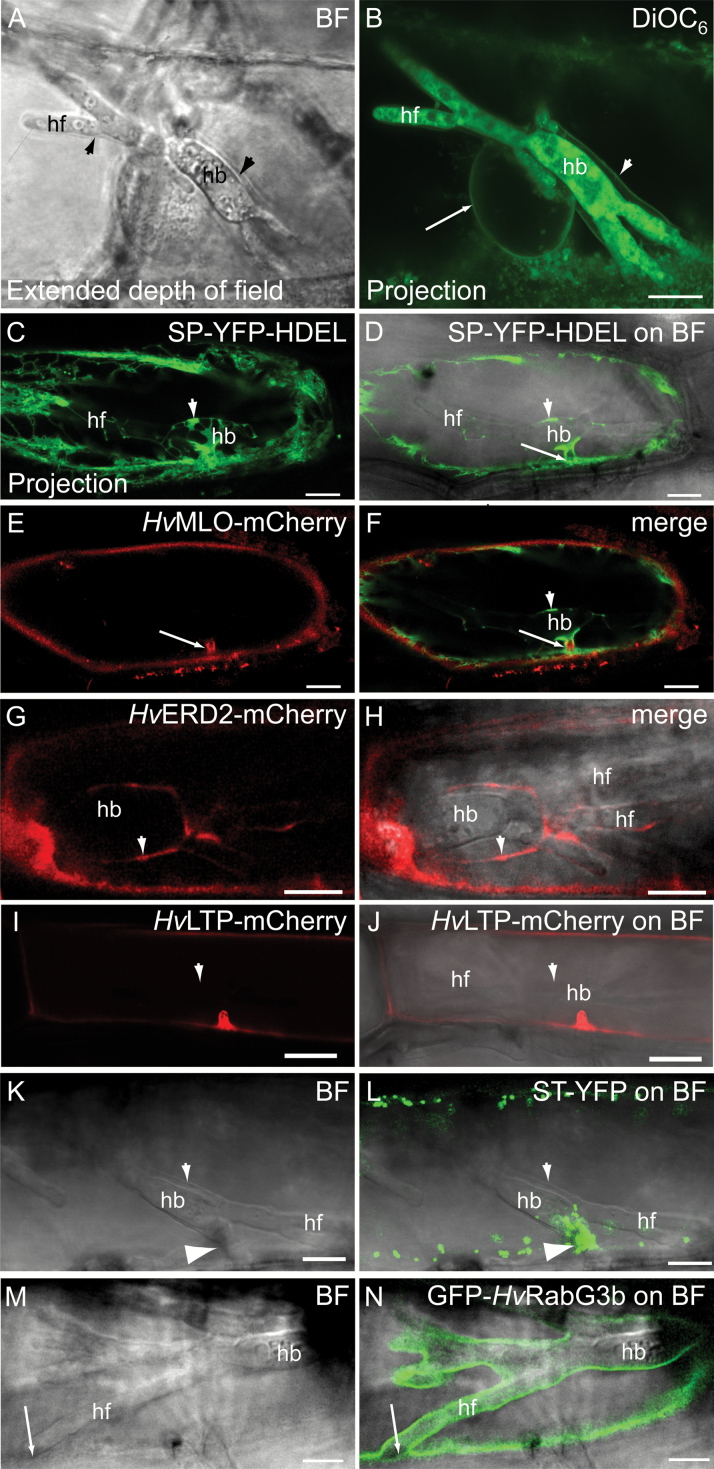
The extrahaustorial membrane, ER-membrane binding dyes and close association with the early secretory pathway. All micrographs show barley epidermal cells containing *Bgh* haustoria at 2 dai. (A, B) Cell stained with 10 μM DiOC_6_. (A) Bright field (BF) image showing the haustorial body, haustorial fingers and the EHM (arrowheads). (B) Maximum intensity projection of the DiOC_6_ fluorescence. Note labelling of the nuclear membrane ER membrane around the nucleus (arrow) and membrane around the haustorium (arrowhead). A single section of this projection can be seen in Supplementary Fig. S2D. (C–F) Cell co-expressing the PM protein *Hv*MLO–mCherry and the ER marker SP–YFP–HDEL. Arrow: haustorial neck; arrowhead: ER tubules and cisternae around the haustorial body. (C) Maximum intensity projection of the SP–YFP–HDEL signal. This micrograph visualizes the ER tubules around the haustorial body. (G, H) Cell expressing the *Hv*ERD2–mCherry marker. Arrowhead: ER at haustorial body. More images of this cell can be seen in Supplementary Fig. S4D–H. (I, J) Cell expressing the PM protein *Hv*LTP–mCherry. Arrowhead: EHM. (K, L) Cell expressing the ST–YFP Golgi marker. Note the accumulation of Golgi bodies at the haustorial neck (arrowhead). (M, N) Cell expressing the tonoplast protein GFP–*Hv*RabG3b. Note that GFP–*Hv*RabG3b stays closely associated with the haustorium, but retracts with the tonoplast at the tips of the haustorial fingers when these are close to the host cell circumference (arrow). This latter observation has been made at least seven times, including those shown in Figs 2 and 3 below. hb: haustorial bodies; hf: haustorial fingers. Scale bars: 10 μm.

Raw image data was exported from the LAS AF lite software package and imported for processing in ImageJ (versions 1.45–1.48, NIH, USA). Using the ‘Window/Level’ function, the whole image was adjusted to use the complete grey scale range without or with a limited amount of saturated areas in the image. This adjustment allowed evaluation of the co-localization of signals independent of the overall fluorophore-dependent signal intensity. Subsequently, the images were blurred, using the ‘Gaussian Blur’ filter with a sigma radius of 1 to reduce background noise, and merged. False colors were assigned to the channels to evaluate co-localization. Maximum intensity projections of a series of consecutive z-stacks were performed using the Zprojection function in ImageJ. The extended depth of field projection shown in Fig. 1A was generated using the ‘Extended depth of field’ plug-in described in Forster *et al.* (2004) (http://bigwww.epfl.ch/demo/edf/index.html, last accessed 15 November 2017).

## Results

### Membrane dyes suggest the EHM surrounding *Bgh* haustoria shares features with the ER membrane

In leaf epidermal cells of the barley host, the *Bgh* fungus forms an elaborate haustorium with a central body and finger-like protrusions, all surrounded by the EHM (Fig. 1A). Previous findings suggests that the host cell ER resides closely to the EHM (Leckie *et al.*, 1995; Koh *et al.*, 2005; Micali *et al.*, 2011; Zhang *et al.*, 2013). By transiently transforming single barley leaf epidermal cells with a luminal ER marker construct, encoding YFP with an N-terminal signal peptide and a C-terminal ER retention signal (SP–YFP–HDEL), we were able to confirm these findings (Fig. 1C–F). YFP-labeled ER tubules and cisternae localized to the fungal entry site and around the haustorial body, but notably less so around the haustorial fingers. Next, we cloned the barley homologue of ER RETENTION DEFICIENT 2 (*Hv*ERD2) and genetically fused it to mCherry. ERD2 is the transmembrane receptor for recycling of proteins containing the ER retention signal H/KDEL. ERD2 shuttles between ER and Golgi via COPII- and COPI-coated vesicles, and it generally marks ER exit sites (ERESs) and Golgi (Hanton *et al.*, 2007, 2008). *Hv*ERD2 showed a similar pattern in relation to haustoria as the luminal ER marker, by being patchy around the haustorial body (arrowhead) and showing less signal around the haustorial fingers (Fig. 1G, H).

To study the ER–EHM association further, we used three dyes that all label the ER membrane: hexyl rhodamine B (Grabski *et al.*, 1993; Zheng *et al.*, 2005; Kankanala *et al.*, 2007; Fitzgibbon *et al.*, 2010; Zhao *et al.*, 2010; Haslam *et al.*, 2012), ER-Tracker Blue-White DPX (Ashtamker *et al.*, 2007) and DiOC_6_ (Terasaki and Reese, 1992; Grabski *et al.*, 1993; Zheng *et al.*, 2004; Martens *et al.*, 2006; Fitzgibbon *et al.*, 2010). As expected, these dyes labelled a membrane around the nucleus, in agreement with their binding to the ER membrane (Fig. 1B and Supplementary Fig. S2A), but also intracellular fungal haustorial membranes (Fig. 1B and Supplementary Fig. S2B–D). All three dyes stained a continuous membrane proximal to the haustorium, which surrounded both the haustorial body and fingers. As the three dyes have the ER membrane as common target, our observations gave the first indication that the EHM may share features with this host membrane. Leckie *et al.* (1995) already showed data suggesting DiOC_6_ stains the EHM. However, it is challenging to separate the EHM from the tonoplast, as these are very close along most of the haustorium, and yet by focusing on cells in which the nucleus is close to the haustorium, Inada *et al* (2016) succeeded in discerning the EHM. A closer look at the DiOC_6_ signal in a single section of the z-stack in Fig. 1B (see Supplementary Fig. S2D) showed that DiOC_6_ stained the membrane between the haustorium and nucleus (diamond in Supplementary Fig. S2D). Furthermore, neither DiOC_6_ nor the two other dyes is reported to stain the tonoplast. If DiOC_6_ would stain the tonoplast, we would have expected to see this by a lumen of cytosol where the nucleus and the haustorium meet in Fig. 1B and Supplementary Fig. S2D. Therefore, the continuous DiOC_6_ stain around the haustorium is suggested to be at the EHM, and in fact we believe that the diamond in Supplementary Fig. S2D marks both the nuclear membrane and the EHM. However, confocal microscopy cannot reveal whether there in fact are two membranes at this site.

### Major plant organelle markers are not found on the EHM

We then investigated in detail how other abundant membranes were positioned in the cell in relation to the haustorium. For this purpose we introduced several well-described cellular markers for the PM, Golgi, and tonoplast. The PM markers, *Hv*MLO–mCherry and *Hv*LTP–mCherry (Kwaaitaal *et al.*, 2010), remained in the PM and did not localize to the EHM (Fig. 1E, F, I, J). However, they were deposited at the haustorial neck, and here *Hv*MLO–mCherry was surrounded by the plant ER (Fig. 1E, F, I, J). The Golgi was visualized using a fusion of the 52-amino-acid signal anchor of a rat sialyltransferase (ST) to YFP (Brandizzi *et al.*, 2002), which accumulated at the fungal entry site (Fig. 1K, L) as previously observed in Arabidopsis (Koh *et al.*, 2005). As for the PM markers, the ST–YFP signal did not extend to the EHM. To visualize the tonoplast, we cloned the barley Ras-related small GTP-binding protein most closely related to *At*RabG3b (see Supplementary Figs S1 and S3A), which is located at the tonoplast in Arabidopsis (Rutherford and Moore, 2002; Vernoud *et al.*, 2003; Carter *et al.*, 2004). A fluorescent fusion, GFP–*Hv*RabG3b, of this Rab GTPase also localized to the tonoplast in barley (Supplementary Fig. S3B–D). In invaded cells, GFP–*Hv*RabG3b surrounded the haustorium (Fig. 1M, N). However, since the tonoplast and the EHM are very close (Inada *et al.*, 2016), confocal microscopy has difficulty in revealing directly to which membrane this marker was associated (see more details below). Therefore, we searched for sites where the haustorial fingers approached the cellular circumference. Here, we detected that GFP–*Hv*RabG3b retracted from the EHM and instead followed the vacuole. Only a weak signal, presumed to derive from the soluble fraction of this fluorescent GTPase, remained at the tip of the haustorial finger (Fig. 1M, N). This situation has been observed in at least seven cases, including this one and the two cases below. In conclusion, none of the shown markers for the tonoplast, PM, and Golgi localized to the EHM.

### Members of the *Hv*Sar1 family of small GTPases localize to the EHM

**Fig. 2.**
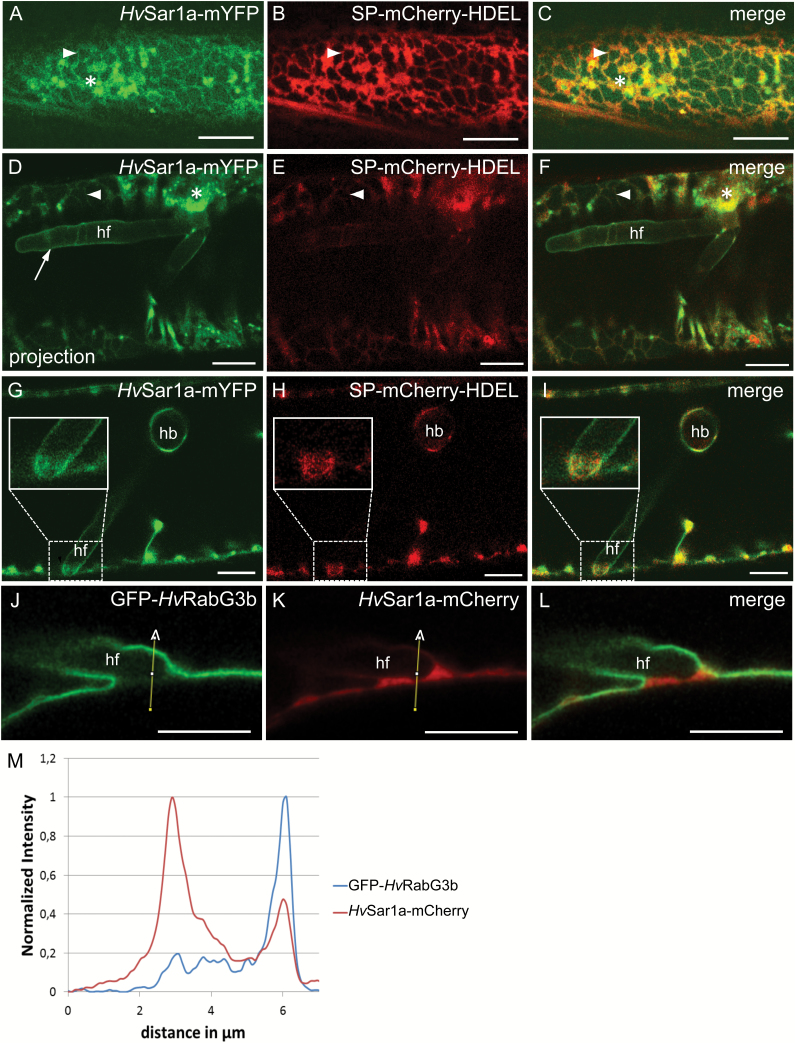
The small GTPase *Hv*Sar1a localized to the EHM. (A–C) Barley epidermal cell co-expressing *Hv*Sar1a–mYFP and the ER luminal marker SP–mCherry–HDEL. (D–L) Barley epidermal cells containing *Bgh* haustoria at 2 dai. (D–I) Cell at two focal planes, (D–F) and (G–I), co-expressing *Hv*Sar1a–mYFP and the ER marker SP–mCherry–HDEL. *Hv*Sar1a–mYFP accumulated at ERESs at the fungal entry site, and at EHM (arrow). Note that *Hv*Sar1a–mYFP remained associated with the EHM at the tip of haustorial fingers near the host cell circumference (insets in G–I). (J–L) Cell co-expressing GFP–*Hv*RabG3b and *Hv*Sar1a–mCherry. (M) Signal quantification of GFP–*Hv*RabG3b and *Hv*Sar1a–mCherry along the indicated path in (J, K). hb: haustorial bodies; hf: haustorial fingers; asterisk: ERES; arrowhead: ER tubules. Scale bars: 10 μm.

We have observations that the EHM can be stained by the ER membrane dyes. However, the ER integral membrane protein marker ERD2 did not label the EHM, nor did the ER luminal marker label the extrahaustorial matrix. This suggested that the EHM may have ER-like properties without being directly connected to the host cell ER. Therefore, we turned to monitor proteins that become associated with the ER membrane from the cytosolic side, such as members of the Sar1 family. These small GTPases bind to the ER membrane by means of an amphipathic domain (Lee *et al.*, 2005), and they are involved in anterograde transport from the ER, where they control COPII-coated vesicle formation from ERESs (Memon, 2004). Barley has three Sar1 isoforms (Böhlenius *et al.*, 2010; Supplementary Fig. S1), which we cloned to make C-terminal fusions to either monomeric (m)YFP or mCherry. Co-localization with the luminal ER marker SP–mCherry–HDEL and a Golgi marker, consisting of the cytoplasmic domain and the transmembrane domain of soybean α-1,2-mannosidase I fused to mCherry (Nelson *et al.*, 2007), revealed that *Hv*Sar1a–mYFP localized to the cytosol, ER-tubules and bright fluorescent structures associated with the ER, representing ERESs (Fig. 2A–C and Supplementary Fig. S4A–C).

Next, we localized *Hv*Sar1 in barley cells containing a haustorium 2 d after inoculation with *Bgh*. Large numbers of *Hv*Sar1a–mYFP-labeled ERESs accumulated at the fungal entry site (Fig. 2D), suggesting active polarized COPII-mediated secretion at this location. In contrast to the ER luminal and transmembrane markers (Zhang *et al.*, 2013) (Fig. 1C, D, F–H), *Hv*Sar1a–mYFP exhibited a continuous signal around fungal haustoria (Fig. 2D–I and Supplementary Fig. S4F–H). The signal intensity of *Hv*Sar1a–mYFP on the EHM is comparable to that on the non-ERES parts of the ER and markedly weaker than that of ERESs (Fig. 2D). *Hv*Sar1b and *Hv*Sar1c exhibited a similar localization to the EHM in *Bgh*-invaded cells as *Hv*Sar1a when fused to mCherry or mYFP (see Supplementary Fig. S4I–N). At sites where the haustorial fingers approached the cellular circumference, *Hv*Sar1a–mYFP remained associated with the haustorium (Fig. 2G–I). At this site, where the tonoplast, the PM and the EHM met, a cytosolic space occurred in which ER structures also accumulated (Fig. 2H). Yet, a continuous *Hv*Sar1a–mYFP signal, which could be distinguished from an ER signal, was present around the tip of the haustorial finger (Fig. 2G–I).

These observations suggested the ER-associated *Hv*Sar1a–mYFP, and not the tonoplast-associated GFP–*Hv*RabG3b (Fig. 1N), labels the EHM. This was further tested by co-localizing GFP–*Hv*RabG3b and *Hv*Sar1a–mCherry. Again GFP–*Hv*RabG3b followed the tonoplast where a haustorial finger was near the PM (Fig. 2J, L). A minor signal, quantified in the intensity plot, between the haustorium and PM is considered to represent the cytosolic portion of this GTPase (Fig. 2J, M), similar to what was seen in Fig. 1N. Also here, the *Hv*Sar1 signal was observed around the haustorial finger. Yet, whether the *Hv*Sar1–mCherry signal in the cytosolic space at the fingertip was soluble, associated with the EHM, with the ER, or all three, could not be distinguished (Fig. 2K, M), unlike in Fig. 2G–I. We could furthermore observe that GFP–*Hv*RabG3b, but not *Hv*Sar1a–mCherry, was excluded from sites where the cell nucleus and haustoria are in close contact (see Supplementary Fig. S5A–E), and that *Hv*Sar1a–mCherry is closer to the haustorium than GFP–*Hv*RabG3b at specific sites (Supplementary Fig. S5F–I). Collectively, these observations indicate that the tonoplast marker, GFP–*Hv*RabG3b, did not label the EHM, while they suggested that the Sar1 markers did.

We subsequently cloned the barley version of Sec12 (see Supplementary Fig. S1) and genetically fused it to mCherry. Sec12 is the ER membrane-localized guanine nucleotide exchange factor (GEF) activating Sar1 (daSilva *et al.*, 2004). As expected, *Hv*Sec12–mCherry co-localized with *Hv*Sar1a–mYFP at the ER tubules (Supplementary Fig. S6A–C). Furthermore, the ER marker *Hv*ERD2–mCherry partially co-localized with *Hv*Sar1a–mYFP at ERESs (Supplementary Fig. S6D–F). These observations confirm an authentic localization of *Hv*Sar1a to ER and ERESs. Sar1, Sec12 and ERD2 all act in COPII vesicle formation (Hanton *et al.*, 2007). As opposed to the Sar1 GTPases, it is noteworthy that *Hv*ERD2–mCherry did not label the EHM above background level (Fig. 1G, H and Supplementary Fig. S4D–H). The localization of *Hv*Sec12–mCherry was less conclusive as we did detect a weak signal following the EHM (Supplementary Fig. S6G–I). Combined, this shows that while the localization of *Hv*Sar1a to the EHM suggests that this membrane indeed has ER-like properties, the absence of *Hv*ERD2 on the EHM indicates that the ER and the EHM do not form a continuous entity, in agreement with the absence of ER luminal markers. Moreover, lack of *Hv*ERD2 at the EHM would suggest that neither COPI- nor COPII-mediated trafficking is directed to or from the EHM.

### The small GTPase *Hv*RabD2a localizes to the EHM

**Fig. 3.**
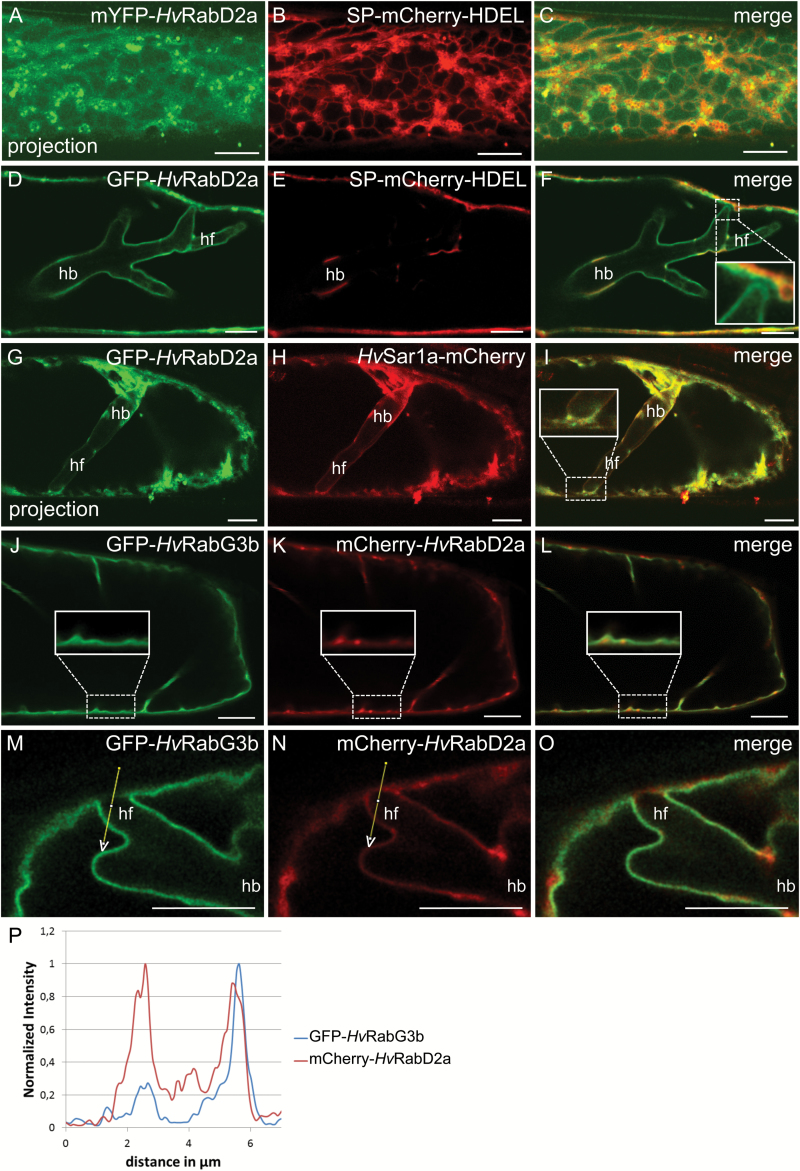
The small GTPase *Hv*RabD2a localized to the EHM. Non-invaded (A–C, J–L) and *Bgh* haustorium-containing (D–I, M–O) barley epidermal cells at 2 dai. (A–F) Barley epidermal cells co-expressing mYFP–*Hv*RabD2a (A, C) or GFP–*Hv*RabD2a (D, F) and the ER luminal marker SP–mCherry–HDEL. Note that GFP–*Hv*RabD2a remains associated with the EHM at the tip of haustorial fingers near the host cell circumference (insert in F). (G–I) Cell co-expressing GFP–*Hv*RabD2a and *Hv*Sar1a–mCherry. (J–O) Barley cells co-expressing GFP–*Hv*RabG3b and mCherry–*Hv*RabD2a. Note that mCherry–*Hv*RabD2a, but not GFP–*Hv*RabG3b, appears associated with the EHM at the tip of haustorial fingers near the host cell circumference. (P) Signal quantification of GFP–*Hv*RabG3b and mCherry–*Hv*RabD2a along the indicated path in (M, N). Images in (A–C) and (G–I) are maximum intensity projection of a series of z-sections. hb: haustorial bodies; hf: haustorial fingers. Scale bars: 10 μm.

The mammalian Rab1 GTPase associates with tethering proteins essential for trafficking of both COPI- and COPII-coated vesicles and, by this, regulates both antero- and retrograde trafficking between the ER and Golgi (García *et al.*, 2011). Likewise, members of the homologous RabD1 and RabD2 clades in Arabidopsis are essential for protein transport from the ER to the Golgi, and localize at the Golgi and the TGN (Batoko *et al.*, 2000; Pinheiro *et al.*, 2009). Rab GTPases in general bind to membranes via lipidation (Barr, 2013). We isolated the closest barley homologue of *At*RabD2a (see Supplementary Figs S1 and S3A) and generated N-terminal fusions with GFP and mYFP. When co-expressed with the soluble ER marker SP–mCherry–HDEL, a spot-like distribution was observed of mYFP–*Hv*RabD2a in association with the ER (Fig. 3A–C). Co-localization analysis with a Golgi marker and the ERES/*cis*-Golgi marker *Hv*ERD2–mCherry confirmed that *Hv*RabD2a mainly co-localized with the ERES/Golgi (Supplementary Fig. S7A–F), as previously observed by Pinheiro *et al.* (2009). The remaining spots labelled by GFP–*Hv*RabD2a that did not co-localize with the Golgi marker likely label the TGN, similarly to its *At*RabD2a homologue in Arabidopsis (Pinheiro *et al.*, 2009). GFP–*Hv*RabD2a and *Hv*Sar1a–mCherry completely co-localized at the ER and ERESs (Supplementary Fig. S7G–I).

In cells invaded by *Bgh*, the GFP–*Hv*RabD2a signal, unlike the ER and Golgi markers, and like the *Hv*Sar1 fluorescent protein fusions, localized around the haustorium (Fig. 3D–I). As suggested for *Hv*Sar1 (Fig. 2G–I), at sites where haustorial fingers approached the cellular circumference (Fig. 3F, inset), the GFP–*Hv*RabD2a signal remained associated with the haustorium. Here, the GFP–*Hv*RabD2a signal at the haustorial fingertip was clearly separated from other signals nearby, indicating that this GTPase is associated with the EHM. Next, in cells in which GFP–*Hv*RabD2a and *Hv*Sar1a–mCherry were co-expressed, these two fluorescently tagged GTPases showed highly overlapping signals around the haustorium and elsewhere in the cell (Fig. 3G–I). Also in this case the signals appeared to follow the EHM around the haustorial fingertip near the cell PM to the extent that this could be distinguished from signals from other structures, potentially the ER (Fig. 3I, inset). As above, when mCherry–*Hv*RabD2a was co-expressed with the tonoplast marker, GFP–*Hv*RabG3b, it was confirmed that these two Rab GTPases associate with separate membranes in control cells (Fig. 3J–L). Here, it was clear that both had a significant cytosolic fraction. In cells harboring a *Bgh* haustorium, this distinct membrane labelling was confirmed, and like in the case of *Hv*Sar1a (Fig. 2J–M), the signal of *Hv*RabD2a between the haustorium and the PM might originate from EHM, ER and cytosolic fractions (Fig. 3M–P).

### *Hv*RabD2a, wild-type and GDP-bound *Hv*Sar1a have a low diffusion rate at the EHM

The finding that the signal intensity of *Hv*Sar1a at the EHM is comparable to the one at non-ERES ER domains might suggest that *Hv*Sar1a binds to the EHM in its GDP-bound state. To verify this observation, we generated an inactive, dominant-negative (DN) GDP-locked mutant, *Hv*Sar1aT34N–mYFP, as described by Ward *et al.* (2001). This DN version of *Hv*Sar1a localized as expected to ER tubules, like wild-type *Hv*Sar1a, but it did not accumulate at ERESs. It furthermore prevented the Golgi α-1,2-mannosidase marker (Nelson *et al.*, 2007) from trafficking from the ER to the Golgi (see Supplementary Fig. S8A–C), as previously shown by Ward *et al.* (2001). This was unlike when the Golgi marker was co-expressed with wild-type Sar1a (Supplementary Fig. S4A–C). This documented the dominant-negative functionality of *Hv*Sar1aT34N–mYFP. Our prediction of GTP-independent binding of Sar1a to the EHM was confirmed using *Hv*Sar1aT34N–mYFP (Supplementary Fig. S8D).

**Fig. 4.**
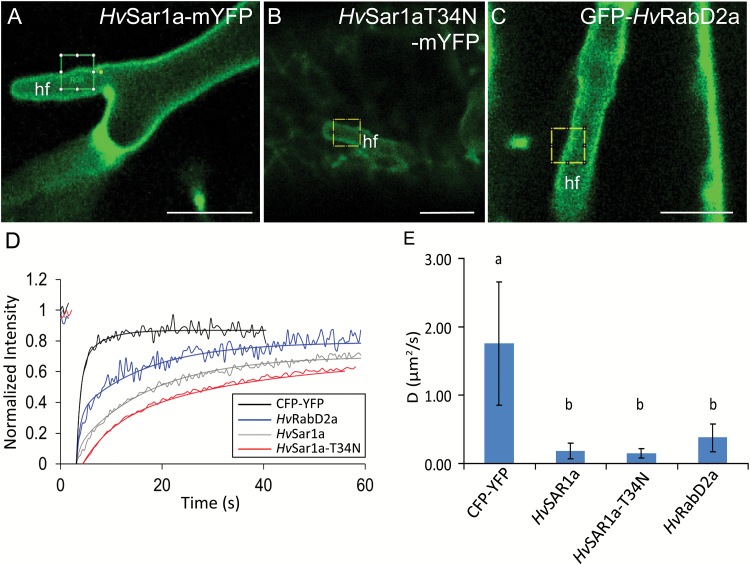
Slow diffusion of *Hv*Sar1a, GDP-locked *Hv*Sar1a and *Hv*RabD2a at the EHM. FRAP measurements of *Hv*Sar1a–mYFP, *Hv*Sar1aT34N–mYFP (GDP-locked), GFP–*Hv*RabD2a, and CFP–YFP expressed in barley epidermal cells containing *Bgh* haustoria at 2 dai. (A–C) Example micrographs for *Hv*Sar1a–mYFP, *Hv*Sar1aT34N–mYFP, and GFP–*Hv*RabD2a, respectively, with the bleached areas marked. Scale bars: 10 μm. (D) Examples of average recovery curves. To allow comparison of the recovery curves, the fluorescence intensity before the bleach was normalized to 1 and at the bleached point to 0. (E) Average diffusion coefficients (*D*) of CFP–YFP and the fluorescent fusion constructs of *Hv*Sar1a–mYFP, *Hv*Sar1aT34N–mYFP, and GFP–*Hv*RabD2a. *D* values were calculated from 11 or more recovery curves originating from three or more biological replicates. hf: haustorial fingers. Error bars show SD. Significance of differences was evaluated using a one-way ANOVA with Tukey’s HSD *post hoc* analysis. The groups a and b differ significantly from one another (*P*<0.01).

We have clearly shown above that, as expected, fluorescent fusion versions of *Hv*Sar1a and *Hv*RabD2a are not associated with the tonoplast. This indicates that the continuous signals around the haustoria from these two GTPases can be from soluble fractions trapped between the EHM and the tonoplast or from a fraction that physically interacts with the EHM, as suggested by the haustorial fingertip observations above. To resolve which of these two fractions provided the signals, we performed FRAP measurements using a soluble cyan fluorescent protein (CFP)–YFP fusion as control. This protein has a similar size as the fluorescent small GTPase fusion proteins. The measured fluorescence recovery rates at the haustorial fingers (Fig. 4A–C) and extrapolated average diffusion coefficients for *Hv*Sar1a–mYFP, *Hv*Sar1aT34N–mYFP and GFP–*Hv*RabD2a were significantly lower than for the cytosolic CFP–YFP (Fig. 4D, E). These recordings were made at areas of the haustoria where no ER was present (Fig. 4A–C). ER was otherwise clearly discernable as bands of more intense signal (Figs 2D–F and 3D–F, and Supplementary Figs S4D–N and S6G–I). This shows that a large proportion of the three versions of the GTPases were temporarily or constitutively membrane-bound at the EHM, and not just freely diffusing in the cytosol.

### *Hv*Sar1- and *Hv*RabD2a-mediated transport appears not essential for EHM formation

Our data indicate that the association of *Hv*Sar1a to the EHM is not dependent on its activation state, suggesting that EHM binding may not have any functional implications, but may be indicative of the physicochemical properties of the membrane. Yet, we followed up on these findings by investigating the potential role of *Hv*Sar1a in mediating membrane transport important for EHM formation by using the GDP-locked version of this GTPase. Expressing this DN GTPase in barley cells did not appear to have any effect on the establishment of *Bgh* haustoria (see Supplementary Fig. S8E), in turn suggesting that EHM formation is not affected and does not require COPII vesicle formation. This observation further suggests that the EHM is generated despite the collapse of the ER and Golgi, as seen when overexpressing *Hv*Sar1aT34N–mYFP (Supplementary Fig. S8A–C). This indirectly suggests that Golgi-formed COPI retrograde vesicles are also not required for EHM formation. To confirm this hypothesis, we made use of the EHM-localized *Hv*RabD2a and analysed whether this GTPase is required for EHM formation. Pinheiro *et al.* (2009) previously demonstrated that the nucleotide-free version of Arabidopsis RabD2a (RabD2aN121I) caused an otherwise secreted protein to be retained in the ER. Expression of *Hv*RabD2aN121I in barley epidermal cells seems not to have an effect on *Bgh* haustorial establishment and thus cannot be linked to EHM formation (Supplementary Fig. S8F).

## Discussion

**Fig. 5.**
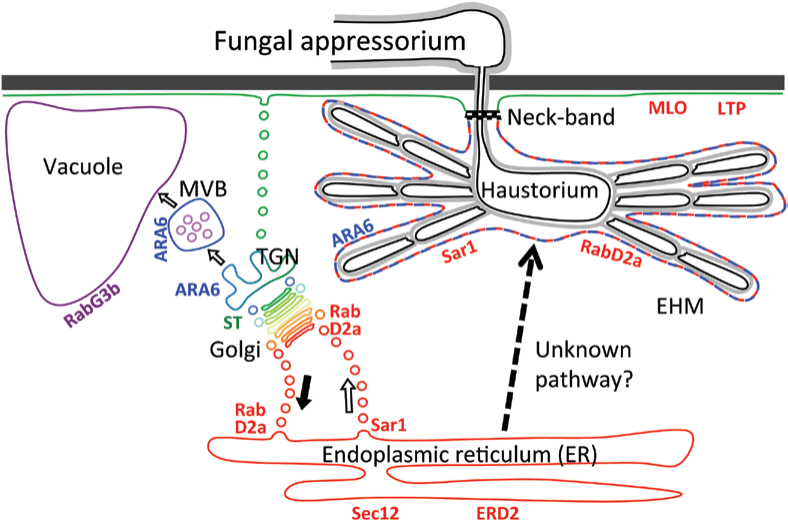
Model localizing protein markers used in this work and suggested pathway for membrane trafficking to the EHM.

Summarized, our data suggested that the EHM stains with ER membrane-binding dyes, which led us to find that it binds the ER-associated proteins *Hv*Sar1 and *Hv*RabD2a. In contrast, ER and Golgi integral membrane and luminal markers failed to accumulate at the EHM or in the extrahaustorial matrix, which suggested that the EHM is directly connected neither to the ER nor to the Golgi. Inada *et al.* (2016) recently demonstrated that the MVB Rab5 GTPase ARA6 binds to the powdery mildew EHM in Arabidopsis and barley. Our results confirm that barley ARA6 is localized around the *Bgh* haustorium (see Supplementary Fig. S9A–C). These observations, together with our suggestion of the EHM having ER properties, points to the EHM being a unique and complex biological membrane, not previously described. A model summarizing our marker localizations is provided in Fig. 5.

COPII-coated vesicle formation at the ER is activated by the Sar1 GEF Sec12, which removes GDP and allows binding of GTP to Sar1 (Futai *et al.*, 2004). GTP binding results in a conformational change of the protein, which exposes an N-terminal amphipathic helix that binds to the ER membrane (Lee *et al.*, 2005). The GDP-locked form of Sar1a still binds ER-enriched microsomes, although with lower affinity than the GTP-locked form. However, it does not sequester the COPII components Sec23/24p and Sec13/31p (Matsuoka *et al.*, 1998). Our images and diffusion measurements suggest that both the wild-type and GDP-locked form of *Hv*Sar1a–mYFP are associated with the EHM. The signal intensity of *Hv*Sar1a–mYFP at the EHM was similar to its intensity at ER tubules and much lower than at ERESs (Fig. 2A–F). These observations combined suggest that Sar1a binds to the EHM with the low, but significant, affinity of the GDP-bound stage. Therefore, its binding is likely due to ER-like properties of the EHM and not related to *Hv*Sar1a activity.

The membrane properties that allow staining with DiOC_6_, hexyl rhodamine B and ER-Tracker Blue-White in plants are poorly described. This is also the case for Rab GTPases (Barr, 2013). Active Sar1a preferentially binds membranes consisting of neutral unsaturated lipids (Matsuoka *et al.*, 1998) and Sar1 GTPase activity is directed to liquid-disordered lipid phases (Long *et al.*, 2010). However, what determines targeting of the GDP-locked *Hv*Sar1a to ER membranes is unknown. Therefore, no suggestions as to the biochemistry of the EHM can be made based on our observations. Yet, our results suggest it is most likely that the ER-like properties of the EHM somehow originate from ER-derived vesicles. Interestingly, the EHM around haustoria of the oomycete *P. infestans* is labelled by a close relative of *Hv*RabG3b (Bozkurt *et al.*, 2015), which we found to be excluded from EHM around powdery mildew haustoria. Even though we cannot rule out that a Rab closely related to *Hv*RabG3b would be able to label the EHM, our data indicate that distinct cellular mechanisms are involved in EHM formation associated with these different pathogens.

One hypothesis to explain the ER-like properties of the EHM is that they are derived from COPI or COPII vesicles. However, our results do not support this hypothesis. The binding of Sar1 to the EHM is independent of GTP, and blocking COPII-coated vesicle budding at the ER by expression of GDP-locked Sar1 did not affect the establishment of haustoria (see Supplementary Fig. S8E). Furthermore, expression of GDP-locked Sar1 most likely indirectly interferes with retrograde COPI vesicle budding from the *cis*-Golgi, since interfering with COPII-coated vesicle formation leads to extensive Golgi stack fragmentation and a gradual loss of Golgi compartments (Osterrieder *et al.*, 2010), as reflected by the retention of the Golgi marker in the ER (Supplementary Fig. S8A–C). Failure of nucleotide-free RabD2aN121I to affect the establishment of haustoria (Supplementary Fig. S8F) appears to support the hypothesis that COPI and COPII vesicles do not provide membrane material for the EHM, since expression of RabD2a with the same mutation in Arabidopsis interfered with ER–Golgi trafficking (Pinheiro *et al.*, 2009). In addition, interference with the ER–Golgi traffic is likely to affect the entire conventional secretory pathway (Takeuchi *et al.*, 2000; Hanton *et al.*, 2008), indicating that membrane needed for the formation of the EHM is not provided by later steps in this pathway. Instead, we suggest that the ER-like properties of the EHM may originate from an unconventional secretory pathway originating from the ER as in the formation of peroxisomes (Hoepfner *et al.*, 2005; Kaur *et al.*, 2009), autophagosomes (Hamasaki *et al.*, 2013; Lamb *et al.*, 2013) or compartments for unconventional protein secretion (Malhotra, 2013). Alternatively, membrane material in the EHM may be derived from direct ER–tonoplast traffic, as suggested by Viotti *et al.* (2013) (see Fig. 5). Such a route might explain why the EHM has both ER properties and late endosome properties, as indicated by the ARA6 labeling (Inada *et al.*, 2016; Supplementary Fig. S9).

Nonetheless, host cell membrane trafficking through the Golgi is important for penetration resistance against *Bgh*. This was demonstrated in barley by Ostertag *et al.* (2013). When they used RNA interference to silence components of the conserved oligomeric Golgi complex COG1 and COG3, the COPIγ coat protein, as well as *Hv*RabD2a, or overexpressed the VTI1-like SNARE protein, the penetration success of *Bgh* was increased. Moreover, they showed that *Hv*RabD2a co-localizes with a Golgi marker and like *Hv*Sar1a–mYFP (Fig. 2D) accumulates below the powdery mildew attack site (Ostertag *et al.*, 2013).

The presence of a novel host membrane with ER-like and MVB-like properties in plant cells harboring fungal haustoria suggests that the pathogen hijacks the host membrane system for its own benefit. Similarly, the human pathogen *Legionella pneumophilla* uses effectors secreted into the host cytosol to redirect ER-derived vesicles to the *Legionella*-containing vacuole (LCV), in which the bacterium replicates. One of the secreted effectors, DrrA, acts as a Rab1 GEF, thereby controlling accumulation of Rab1 on the LCV membrane (Murata *et al.*, 2006). We speculate that *Bgh* introduces effectors into the host cell to control vesicle traffic in a similar way and thereby facilitates the formation of this specialized EHM. The *Bgh* genome encodes hundreds of candidates for effector proteins (Spanu *et al.*, 2010; Pedersen *et al.*, 2012) and future efforts will include attempts to identify effectors controlling EHM generation. Understanding the vesicle trafficking pathway leading to EHM formation and how it may be regulated by effectors will be essential in future plant genome editing approaches aimed at preventing EHM formation and thereby preventing powdery mildew infection.

## Supplementary data

Supplementary data are available at *JXB* online.

Fig. S1. Alignments of barley proteins used in this study to their closest relatives in Arabidopsis.

Fig. S2. ER membrane dyes and the extrahaustorial membrane.

Fig. S3. Barley Rab GTPases.

Fig. S4. Three Sar1 GTPases, but not the HDEL receptor ERD2, labeled the extrahaustorial membrane.

Fig. S5. The tonoplast marker *Hv*RabG3b did not label the EHM.

Fig. S6. Sar1a, ERD2, the Sar1 GEF Sec12, and the EHM.

Fig. S7. RabD2a co-localized with Golgi marker, ERD2 and Sar1a.

Fig. S8. Arresting ER–Golgi traffic did not affect haustorial formation.

Fig. S9. ARA6 labels EHM.

Table S1. Primer sequences.

supplementary-figures-S1-S9 and Table-S1

## Abbreviations:

BFA: brefeldin A
Bgh: *Blumeria graminis* f.sp. *hordei*
EHM: extrahaustorial membrane
ER: endoplasmic reticulum
FRAP: fluorescence redistribution after photobleaching
MVB: multivesicular body
PM: plasma membrane
TGN: *trans*-Golgi network.
