# Supplementary material for: The plant membrane surrounding powdery mildew haustoria shares properties with the endoplasmic reticulum membrane
Source: J Exp Bot. 2017 Dec 9;68(21-22):5731–43. doi: 10.1093/jxb/erx403 (PMC5854130; doi:10.1093/jxb/erx403)
Supplement: supplementary-figures-S1-S9 and Table-S1 [file erx403_suppl_supplementary_figures_s1_s9_table_s1.pdf]

Supplementary data

The plant membrane surrounding powdery mildew haustoria shares properties with the endoplasmic reticulum

Mark Kwaaitaal, Mads Eggert Nielsen, Henrik Böhlenius and Hans Thordal-Christensen

Kwaaitaal et al. Supplementary Figure S1

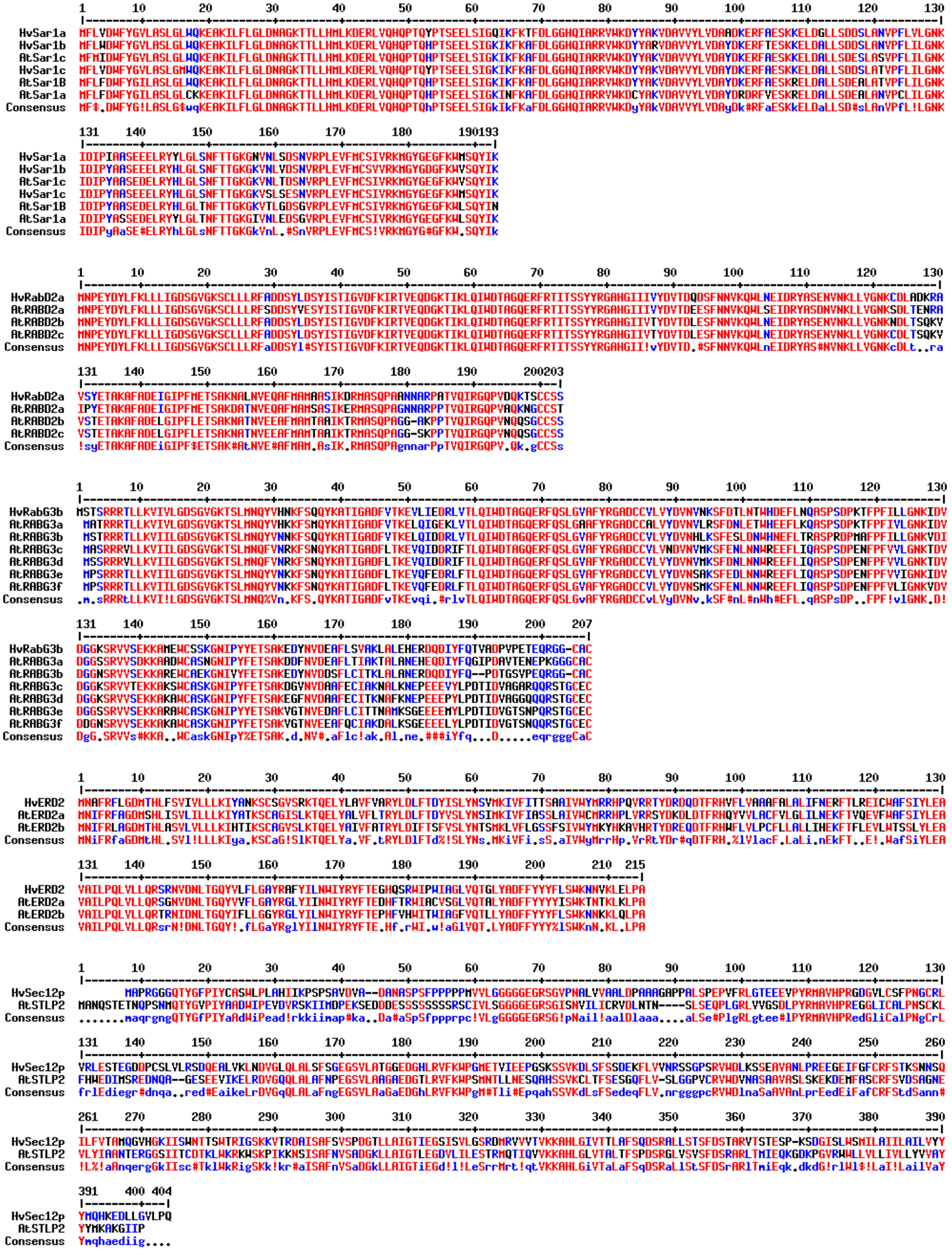

Supplementary Figure S1. Alignments of barley proteins used in this study to their closest relatives in Arabidopsis.

These are, AtSar1a: At1G09180, AtSar1b: At5G56330, AtSar1c: At4G02080, AtRabD2a: At1G02130, AtRabD2b: At5G47200, AtRabD2c: At4G17530, AtRabG3a: At4G09720, AtRabG3b: At1G22740, AtRabG3c: At3G16100, AtRabG3d: AT1G52280, AtRabG3e: AtAt1G49300, AtRabG3f: At3G18820, AtERD2a: At1G29330, AtERD2b: At3G25040, AtSTLP2: At2G01470. The alignments were made using http://multalin.toulouse.inra.fr/multalin/cgi-bin/multalin.pl.

Kwaaitaal et al. Supplementary Figure S2

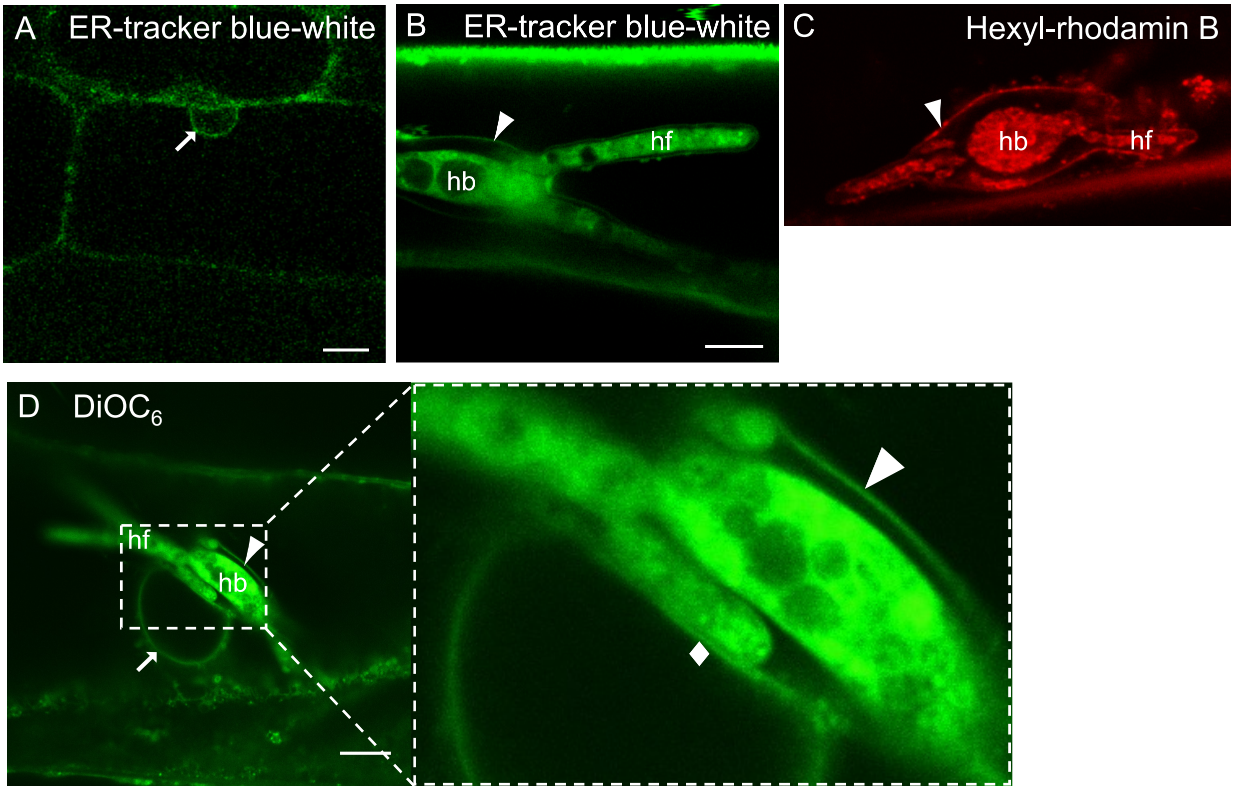

Supplementary Figure S2. ER-membrane dyes and the extrahaustorial membrane.
(A) Non-inoculated barley cell stained with ER-tracker blue-white. (B-D) Barley cells containing *Bgh* haustoria 2 days after inoculation (dai) stained with ER-tracker blue-white (B), Hexyl-rhodamin B (C) and $DiOC_6$ (D). ER membrane around the nucleus (arrows), membrane around haustoria (arrowhead), membrane between nucleus and haustorium (diamond), haustorial bodies (hb), haustorial fingers (hf). Scale bar, 10 µm.

Kwaaitaal et al. Supplementary Figure S3 (1st of 2 pages)

A

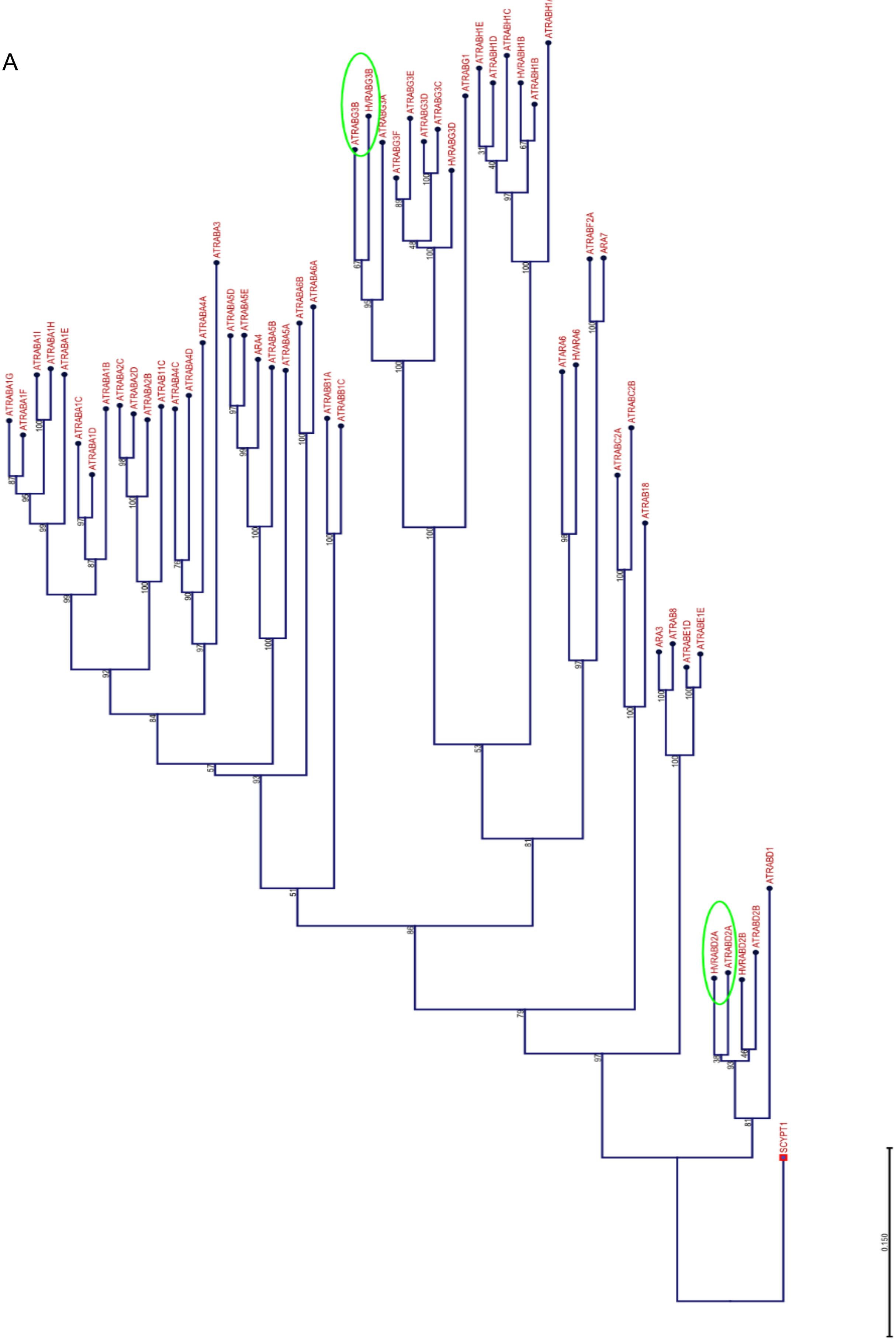

Kwaaitaal et al. Supplementary Figure S3 (2nd of 2 pages)

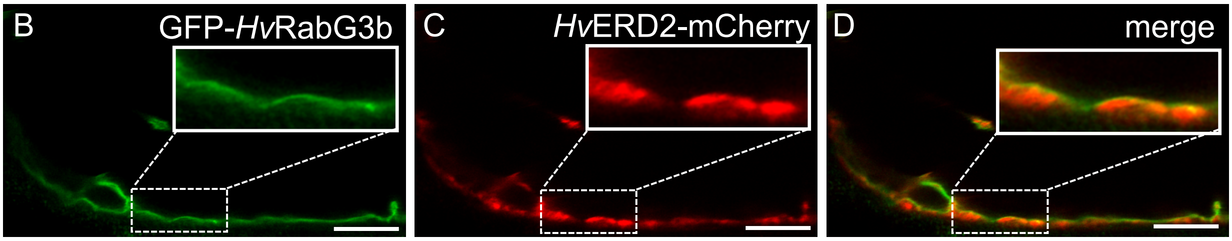

Supplementary Figure S3. Barley Rab GTPases.
(A) Phylogenetic analysis of members of the Arabidopsis and barley Rab GTPase family. Arabidopsis Rab protein sequences were obtained from the Genbank database and named according to Rutherford and Moore (2002) and Vernoud et al. (2003). Yeast *Sc*Ypt1 is used as an outgroup. The barley Rab GTPases were named according to their closest homologues in Arabidopsis. *Hv*RabD2a and *Hv*RabG3b are circled. Protein sequence comparison and subsequent bootstrapping (1000 iterations) was performed with the CLC sequence viewer (Qiagen). (B-D) Micrographs of a non-inoculated barley epidermal cell co-expressing the vacuolar marker GFP-*Hv*RabG3b and the ERES/Golgi marker *Hv*ERD2-mCherry. Inserts show magnified area where GFP-*Hv*RabG3b marks a membrane towards the inside of the cell and *Hv*ERD2-mCherry marked the ERES/Golgi in the cytosol. This observation confirms that GFP-*Hv*RabG3b marked the tonoplast. Scale bar, 10 µm.

Rutherford S, Moore I. 2002. The Arabidopsis Rab GTPase family: another enigma variation. *Current Opinion of Plant Biolology* **5:** 518–528.
Vernoud V, Horton AC, Yang Z, Nielsen E. 2003. Analysis of the small GTPase gene superfamily of *Arabidopsis*. *Plant Physiology* **131:** 1191–1208.

Kwaaitaal et al. Supplementary Figure S4

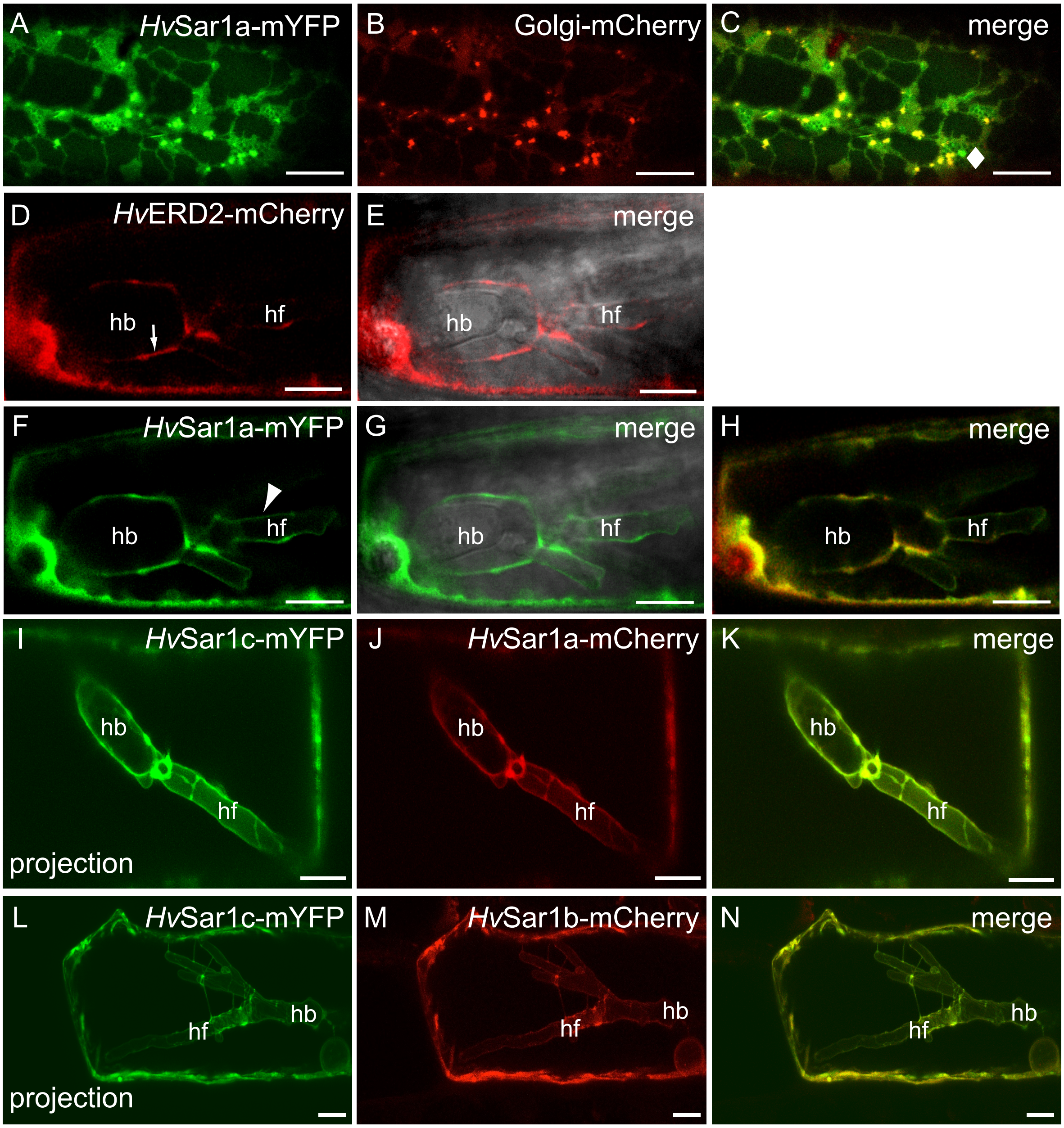

Supplementary Figure S4. Three Sar1 GTPases, but not the HDEL-receptor, ERD2, labeled the extrahaustorial membrane.
(A-C) Non-inoculated barley cell co-expressing *Hv*Sar1a-mYFP with the Golgi-mCherry marker (soybean α-1,2-mannosidase I fused to mCherry). (D-H) *Bgh* haustorium-containing barley cell (2 dai) co-expressing the ERES/cis-Golgi marker, *Hv*ERD2-mCherry, and *Hv*Sar1a-mYFP. Note, *Hv*ERD2 did not labeling the EHM. Note, D and E are also shown in Figure 1, and repeated here for comparison. (I-K) *Bgh* haustorium-containing barley cell (2 dai) co-expressing *Hv*Sar1c-mYFP and *Hv*Sar1a-mCherry. (L-N) *Bgh* haustorium-containing barley cell (2 dai) co-expressing *Hv*Sar1c-mYFP and *Hv*Sar1b-mCherry. Note, localization of *Hv*Sar1b and *Hv*Sar1c at the EHM similar to *Hv*Sar1a. ER (arrow); EHM (arrowhead), ERES not labeled with the Golgi marker (diamond), haustorial bodies (hb), haustorial fingers (hf). Scale bars, 10 µm.

Kwaaitaal et al. Supplementary Figure S5

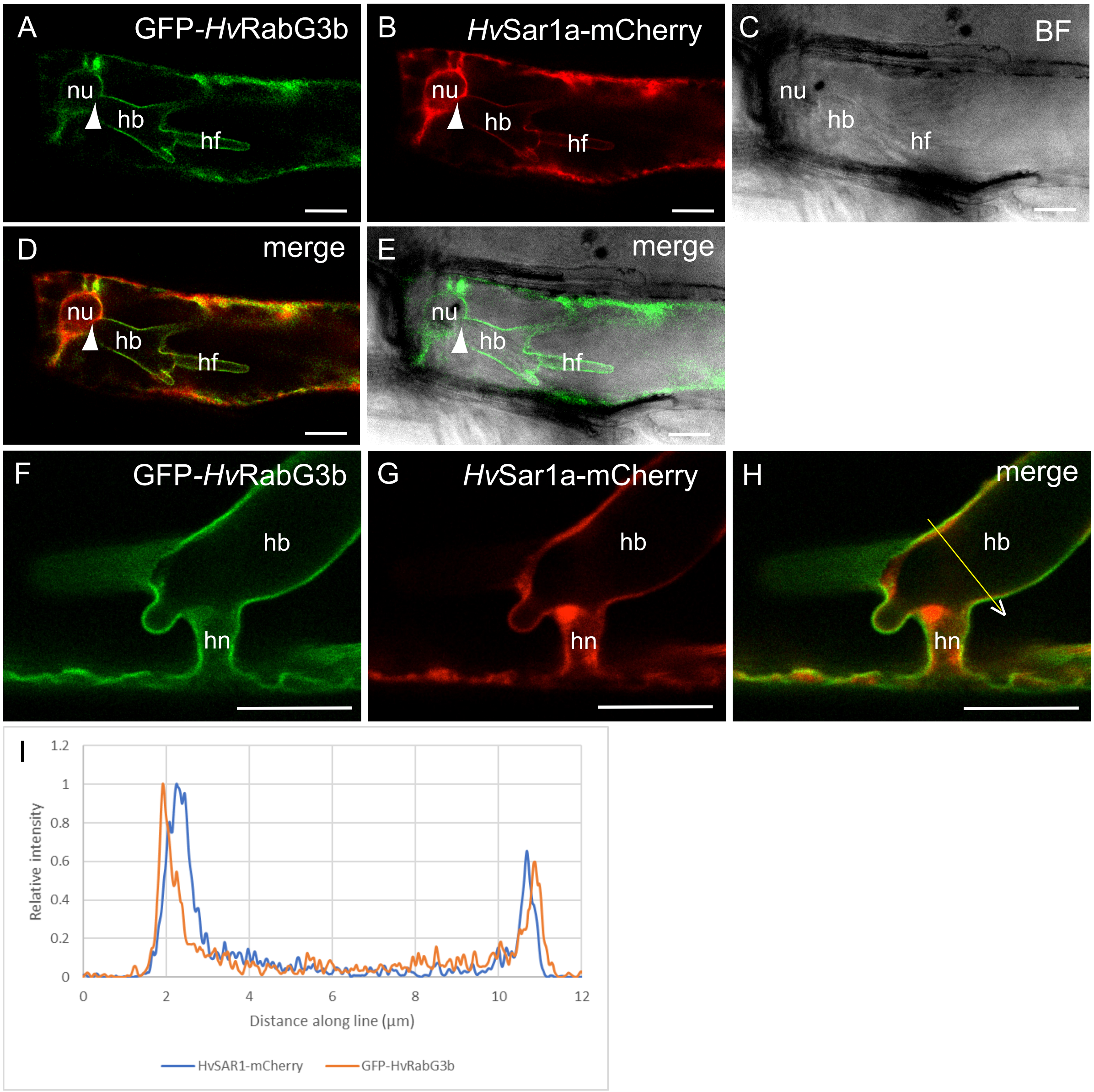

Supplementary Figure S5. The tonoplast marker, *Hv*RabG3b, did not label the EHM. (A-H) *Bgh* haustorium-containing barley cell (2 dai) co-expressing the GFP-*Hv*RabG3b and *Hv*Sar1a-mCherry. (A-E) Note, GFP-*Hv*RabG3b was absent between the nucleus and the haustorial body (arrowhead). (F-I) Note, ER/EHM-associated *Hv*Sar1a-mCherry found between GFP-*Hv*RabG3b and haustorium. (I) Signal quantification of GFP-*Hv*RabG3b and *Hv*Sar1a-mCherry along the indicated path in H. Bright filed (BF), haustorial bodies (hb), haustorial fingers (hf), haustorial neck (hn). Scale bars, 10 µm.

Kwaaitaal et al. Supplementary Figure S6

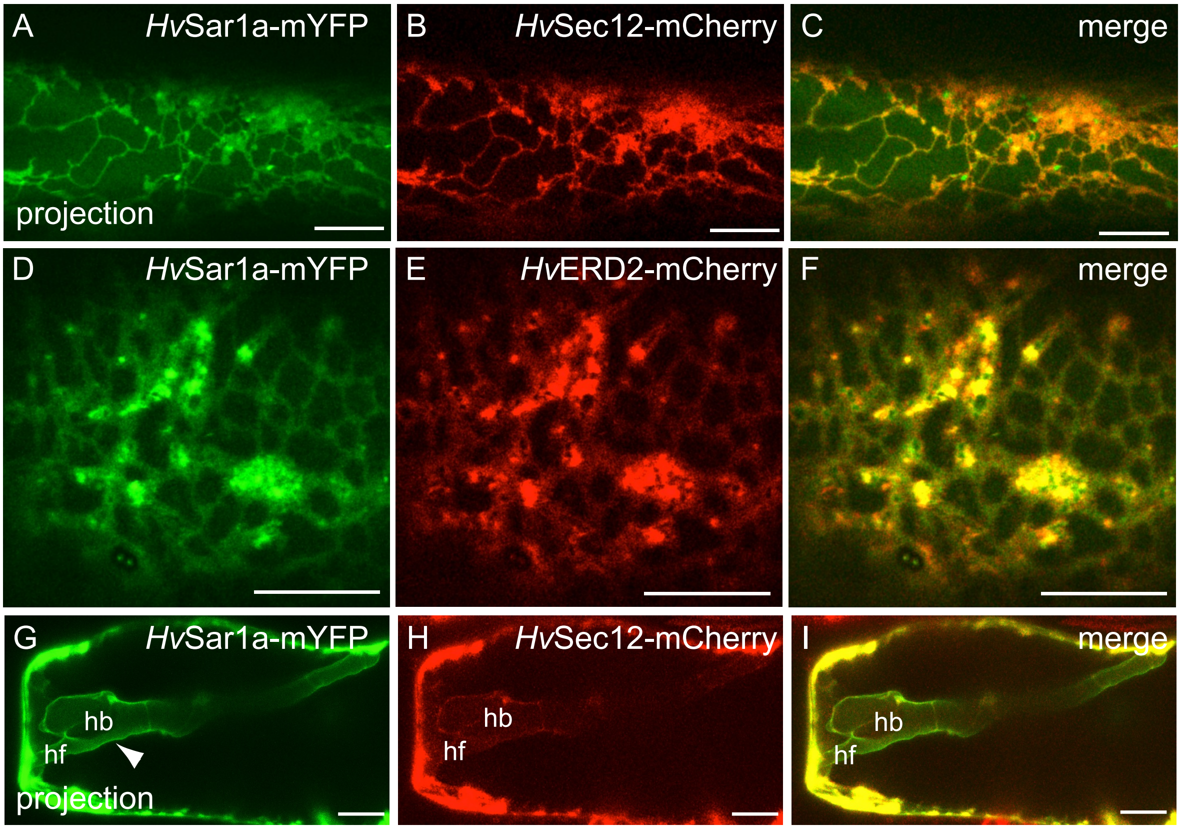

Supplementary Figure S6. Sar1a, ERD2, the Sar1 GEF, Sec12, and the EHM.
(A-C) Barley cells co-expressing *Hv*Sar1a-mYFP and *Hv*Sec12-mCherry. (D-F) Cells co-expressing *Hv*Sar1a-mYFP and *Hv*ERD2-mCherry. (G-I) *Bgh* haustorium-containing barley cell (2 dai) co-expressing *Hv*Sar1a-mYFP and *Hv*Sec12-mCherry. EHM (arrowhead), haustorial bodies (hb), haustorial fingers (hf). Scale bars, 10 µm.

Kwaaitaal et al. Supplementary Figure S7

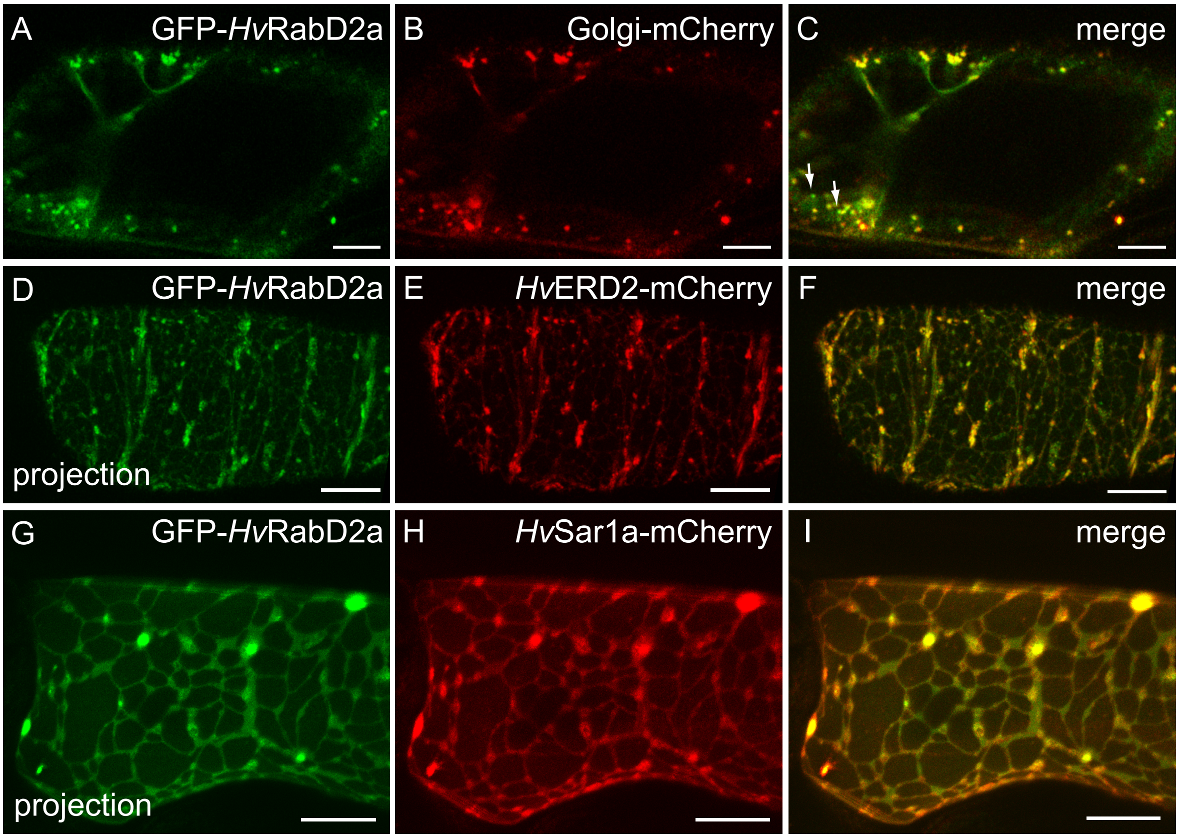

Supplementary Figure S7. RabD2a co-localized with Golgi-marker, ERD2 and Sar1a.

(A-I) Non-inoculated barley cells. (A-C) Barley cell co-expressing GFP-*Hv*RabD2a and the Golgi-mCherry marker (soybean α-1,2-mannosidase I fused to mCherry). Arrows in C mark GFP-*Hv*RabD2a-positive units not labeled as Golgi compartments. (D-F) Barley cell co-expressing GFP-*Hv*RabD2a and *Hv*ERD2-mCherry. (G-I) Barley cell co-expressing GFP-*Hv*RabD2a and *Hv*Sar1a-mCherry. Scale bars, 10 µm.

Kwaaitaal et al. Supplementary Figure S8

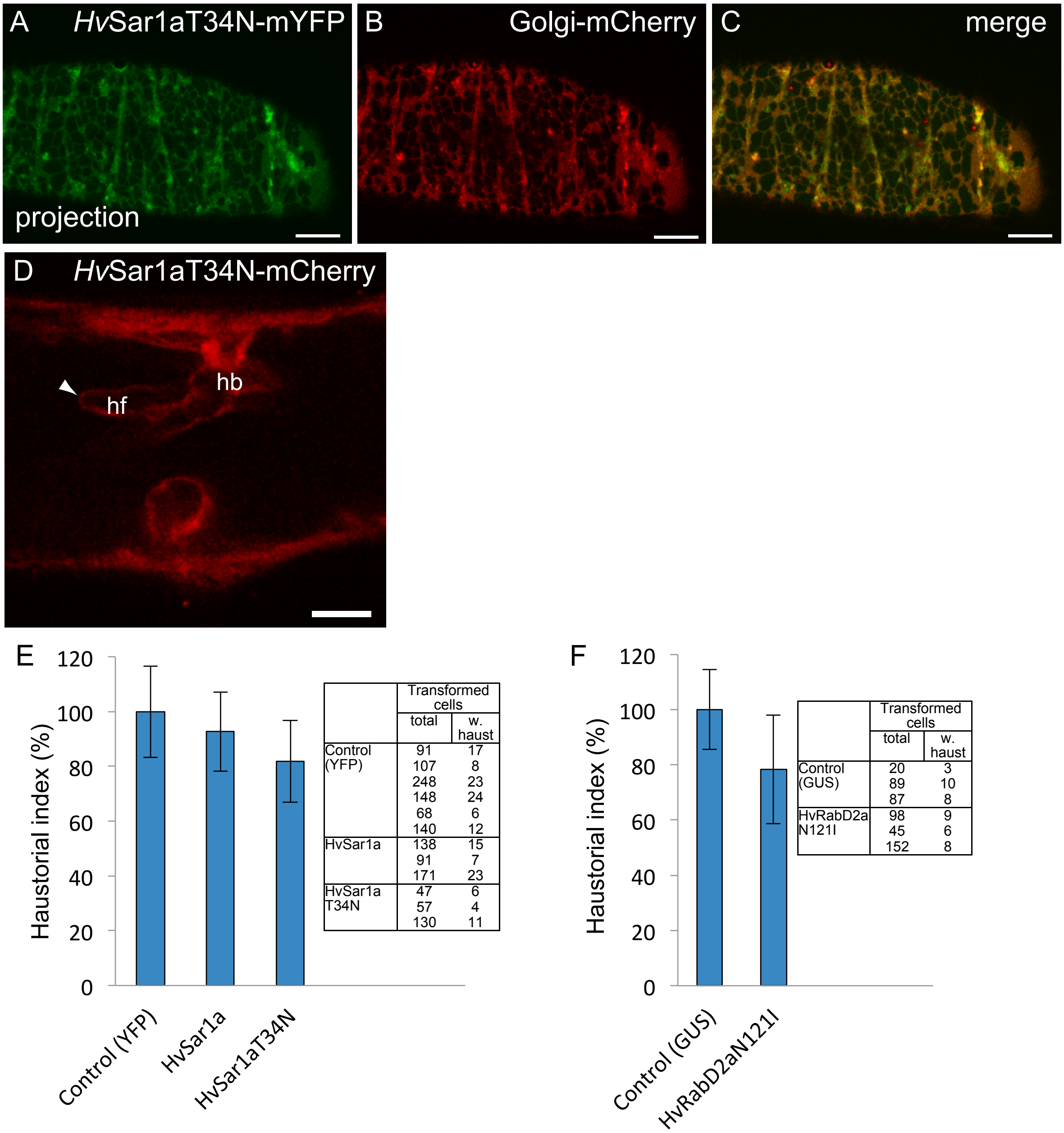

	Transformed cells	
	total	w. haust
Control (YFP)	91	17
	107	8
	248	23
	148	24
	68	6
	140	12
HvSar1a	138	15
	91	7
	171	23
HvSar1a T34N	47	6
	57	4
	130	11

	Transformed cells	
	total	w. haust
Control (GUS)	20	3
	89	10
	87	8
HvRabD2a N121I	98	9
	45	6
	152	8

Supplementary Figure S8. Arresting ER-Golgi traffic did not affect haustorial formation.

(A-C) Non-inoculated barley cell co-expressing *Hv*Sar1aT34N-mYFP (GDP-locked) and the Golgi-mCherry marker (soybean α-1,2-mannosidase I fused to mCherry). Note Golgi-marker was prevented from progressing beyond the ER. (D) *Hv*Sar1aT34N-mCherry labels the EHM in barley cell co-expressing containing *Bgh* haustorium 2 dai. EHM (arrowhead), haustorial bodies (hb), haustorial fingers (hf). Scale bar, 10 µm. (E-F) *Bgh* haustorial formation (2 dai) after overexpression of *Hv*Sar1a and *Hv*Sar1aT34N (GDP-locked) and after expression of *Hv*RabD2aN121I (nucleotide free). Average haustorial indices are provided relative to the average of controls, set to 100%. Error bars show SE. Values are averages of biological replicates, as indicated in the provided raw data.

Kwaaitaal et al. Supplementary Figure S9

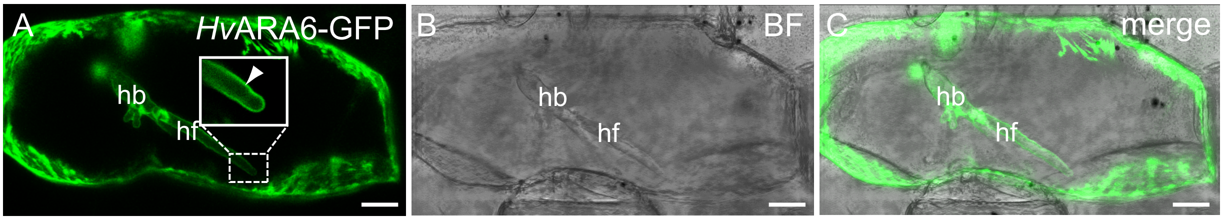

Supplementary Figure S9. ARA6 labels EHM.
(A-C) *Bgh* haustorium-containing barley cell (2 dai) expressing *Hv*ARA6-GFP. Note insert, *Hv*ARA6-GFP associated with EHM. EHM (arrowhead), haustorial bodies (hb), haustorial fingers (hf). Scale bar, 10 µm.

Kwaaitaal et al. Supplementary Table S1

Supplementary Table S1. Primer sequences.

**Primer Name**	**Sequence**
HvSar1a *(Genbank: AK252291.1)*	
Fluorescent fusion	
HvSAR1a_GWY_KZK_F	GGGGACAAGTTTGTACAAAAAAGCAGGCTTAACCATGTTTCTGGTGGATTGGTT
HvSAR1a_GWY_nst_R	GGGGACCACTTTGTACAAGAAAGCTGGGTTCTTGATGTACTGGGACATCCATT
Overexpression	
HvSar1a_Fw	GGGGACAAGTTTGTACAAAAAAGCAGGCTTC ATGTTTCTGGTGGATTGGTTCTA
HvSar1a_Rv	GGGGACCAGTTTGTACAAGAAAGCTGGGTC TCACTTGATGTACTGGGA
HvSar1aT34N	
HvSar1aT34N_F	GACAACGCTGGCAAGAaCACCCTCCTCCACATG
HvSar1aT34N_R	CATGTGGAGGAGGGTGtTCTTGCCAGCGTTGTC
HvSar1b *(Genbank: AK252372.1)*	
Fluorescent fusion	
HvSAR1b_GWY_KZK_F	GGGGACAAGTTTGTACAAAAAAGCAGGCTTAACCATGTTCCTTTGGGACTGGTT
HvSAR1b_ GWY_nst_R	GGGGACCACTTTGTACAAGAAAGCTGGGTTCTTGATGTACTGCGAGACCCACT
HvSar1c *(Genbank: AK250294.1)*	
Fluorescent fusion	
HvSAR1c_GWY_KZK_F	GGGGACAAGTTTGTACAAAAAAGCAGGCTTAACCATGTTCCTTGTGGACTGGTT
HvSAR1c_ GWY_nst_R	GGGGACCACTTTGTACAAGAAAGCTGGGTTCTTAATATACTGCGACATCCACT
HvRabD2a *(Genbank: AK355333.1)*	
HvRABD2a_GWY_F	GGGGACAAGTTTGTACAAAAAAGCAGGCTTCATGAATCCGGAGTATGATTACCTCTT
HvRABD2a_GWY_stop_R	GGGGACCAGTTTGTACAAGAAAGCTGGGTCCTAAGAAGAGCAGCAGCTGGTCTTC
HvRabD2aN121I	
HvRabD2aN121I_F	AAGCTTCTCGTGGGGAtCAAATGTGATCTCGCT
HvRabD2aN121I_R	AGCGAGATCACATTTGaTCCCCACGAGAAGCTT
RabG3b *(Genbank: AK368361.1)*	
HvRabG3b_GWY_F	GGGGACAAGTTTGTACAAAAAAGCAGGCTTCATGGCCTCGCGACGCCGCACGC
HvRabG3b_GWY_R	GGGGACCAGTTTGTACAAGAAAGCTGGGTCCTAACAGCAGCCTGGTGATCTT
HvERD2 (Genbank: AK250768.1)	
HvERD2_F	AATAAACCCCCCAAAACC
HvERD2_R	GCCAATACAGACCATCAA
HvERD2_GWY_KZK_ F	GGGGACAAGTTTGTACAAAAAAGCAGGCTATACCATGAACGCCTTCCGG
HvERD2_GWY _nst _R	GGGGACCACTTTGTACAAGAAAGCTGGGTTGGCAGGCAACTCGAGCTTC
HvSec12 *(Genbank: AK356828.1)*	
HvSec12_F1	TAGTTGTGAACAGGAGCAG
HvSec12_R1	AGAGAGGTAAGCAGTGAAA
HvSec12_GWY_KZK_F	GGGGACAAGTTTGTACAAAAAAGCAGGCTTAACCATGGCGCCGCGCGGCGGTGG
HvSec12_GWY_nst_R	GGGGACCACTTTGTACAAGAAAGCTGGGTTTTGTGGCAACACGCCTAAGA
